# Association between Serum Magnesium and Hemoglobin in Patients with Primary Hyperparathyroidism

**DOI:** 10.1155/2021/6049317

**Published:** 2021-11-27

**Authors:** Na Ding, Tao Guo, Shu-Ying Liu, Qin-Yi Wang, Xiao-Li Qu, Yong-Fang Li, Yang-Na Ou, Yan-Yi Yang, Zhi-Feng Sheng

**Affiliations:** ^1^National Clinical Research Center for Metabolic Diseases, Hunan Provincial Key Laboratory of Metabolic Bone Diseases, Health Management Center and Department of Metabolism and Endocrinology, The Second Xiangya Hospital of Central South University, Changsha 410011, Hunan, China; ^2^Blood Transfusion Department, Zhuzhou Central Hospital, Zhuzhou 412007, Hunan, China; ^3^Department of Surgery, The First Affiliated Hospital of Hunan Normal University, Changsha 410005, Hunan, China; ^4^Hospital Infection Control Center, The Second Xiangya Hospital of Central South University, 139 Middle Renmin Road, Changsha 410011, Hunan, China; ^5^Health Management Center, The Second Xiangya Hospital of Central South University, 139 Middle Renmin Road, Changsha 410011, Hunan, China

## Abstract

**Background:**

There is a positive association between serum magnesium and hemoglobin levels in the general population. However, no studies have evaluated the association between serum magnesium and hemoglobin levels in patients with primary hyperparathyroidism (PHPT). We aimed to investigate whether there is a relationship between serum magnesium and hemoglobin levels in the patient population with PHPT.

**Methods:**

This retrospective study included 307 hospitalized PHPT patients who were continuously admitted to the Second Xiangya Hospital of Central South University, from January 2010 to August 2020. Laboratory and demographic data of patients were collected. Hypomagnesemia was defined as serum magnesium <0.75 mmol/L. Patients with a hemoglobin level below 130 g/L in males and below 120 g/L in females were accepted as the anemic group.

**Results:**

Among the 307 patients with PHPT included in our study, 77 (25.1%) patients (33 (32.4%) males and 44 (21.5%) females) had hypomagnesemia. A total of 138 (45.0%) patients (49 males (48.0%) and 89 females (43.4%)) had anemia. Compared with the nonanemic group, the anemic group had lower average albumin, eGFR, and serum magnesium levels in both males and females. In contrast, average creatinine, PTH, and corrected calcium were significantly higher in the anemic group than in the nonanemic group in both males and females. Lower serum magnesium levels were associated with lower hemoglobin levels independent of serum calcium, albumin, eGFR, and PTH in PHPT patients.

**Conclusions:**

Hypomagnesemia is a common electrolyte disorder in PHPT patients. Hypomagnesemia is independently associated with lower hemoglobin levels in patients with PHPT.

## 1. Introduction

Primary hyperparathyroidism (PHPT) is a common endocrine disease characterized by hypercalcemia and high or inappropriately normal levels of parathyroid hormone (PTH) [1–3]. In PHPT, there is a range of symptoms caused by hypercalcemia that affect the skeletal, renal, and digestive systems [4]. Anemia has been recognized as a possible complication of PHPT [5–7]; however, the exact pathogenesis of anemia is still unknown. It is possible that patients develop anemia when the kidneys do not produce sufficient erythropoietin (EPO) levels to promote erythropoiesis [8]. The bone and bone marrow, although often regarded as separate systems, function as a single unit. The bone marrow contains hematopoietic cells, and bone marrow suppression causes anemia [9]. Anemia is also a common complication of gastrointestinal tract diseases [10]. In addition, high PTH levels may play a role in anemia in PHPT patients. Researchers have reported that EPO synthesis, erythroid progenitor production, and red blood cell (RBC) survival are negatively impacted under high PTH [11–13]. Similarly, high serum levels of PTH negatively affect serum hemoglobin levels in patients with chronic kidney disease (CKD) [14, 15]. There is indirect evidence of restoration of hematocrit levels following control of secondary hyperparathyroidism or parathyroidectomy in uremic patients due to restoration of bone marrow space and increase in immunoreactive EPO levels [16–19].

Magnesium, which is the second most important intracellular cation, has received considerable interest within the scientific community. The relationship between magnesium deficiency and anemia was first reported in animal studies [20, 21]. In 1973, Piomelli et al. observed a significant decrease in hemoglobin levels in rats fed with a magnesium-deficient diet. This anemia was accompanied by severe reticulocytosis, reduction in hemoglobin and hematocrit levels, and decrease in RBC lifespan and half-life. The authors argued that magnesium deficiency contributed to hemolytic anemia by affecting energy metabolism in RBCs [21]. A study conducted with 8,511 participants showed that high magnesium levels were associated with a lower risk of anemia [22]. Similarly, an inverse relationship was reported between magnesium and anemia in the elderly patient population [23]. Positive effects of high serum magnesium on hemoglobin have been reported in CKD patients [24, 25]. It is worth mentioning that magnesium supplementation increases hemoglobin levels in athletes [26].

The characteristics of serum magnesium in PHPT patients are still controversial. A previous study reported that serum magnesium was usually within the normal range in PHPT [27]; however, hypomagnesemia has been noted in PHPT patients, especially in association with very high serum calcium levels or kidney damage [28–30]. Therefore, the characteristics of serum magnesium in patients with PHPT remain unclear. To the best of our knowledge, no study has investigated the association between magnesium and hemoglobin levels in patients with PHPT. In this study, we investigated whether there is an association between serum magnesium and hemoglobin levels in PHPT patients.

## 2. Materials and Methods

### 2.1. Study Design and Patients

We performed a retrospective study of 307 hospitalized patients with PHPT, who were admitted to Second Xiangya Hospital of Central South University, which is a tertiary hospital in Changsha, Hunan Province, Central South of China, from January 2010 through August 2020. Patients diagnosed with secondary hyperparathyroidism, tertiary hyperparathyroidism, familial hypocalciuric hypercalcemia (FHH), osteitis fibrosa cystica (OFC), or gastrointestinal bleeding were excluded from the study. Hypomagnesemia was defined as serum magnesium <0.75 mmol/L [31]. Anemia was defined as hemoglobin <130 g/L in males and <120 g/L in females [32].

This study protocol was approved by the Ethics Committee. All the patients provided informed consent for participating in this study.

### 2.2. Medical History Collection and Anthropometric Information

Medical records were reviewed for age, sex, and disease duration. The patient height was measured to the nearest 0.1 cm, and the weight was recorded to the nearest 0.1 kg with the participant wearing light clothing. BMI was calculated as weight in kilograms divided by height in meters squared.

### 2.3. Biochemical Measurements

We recorded the levels of serum calcium, phosphorus, magnesium, albumin, creatinine, PTH, and 25(OH)D. Blood samples were collected after an overnight fast. The measurement of hemoglobin (Hb) levels was performed by using the automated hematology analyzer ADVIA 2120 (Siemens Healthcare Diagnostics, Germany). Serum albumin, calcium, phosphorus, and magnesium were determined using an automatic biochemical analyzer (Abbott Laboratories, North Chicago, IL, USA). Albumin-corrected serum calcium was calculated using the following formula, corrected calcium (mmol/L) = serum calcium ((mmol/L) + 0.02  ×  (40 − serum albumin (g/L)). Serum creatinine levels were determined by an enzymatic method (Kanto Chemical, Tokyo, Japan), and the estimated glomerular filtration rate (eGFR) was quantified using CKD-EPI 2009 equations [33]. Serum PTH was measured by automated chemiluminescence immunoassay (Siemens Healthcare Diagnostics, Erlangen, Germany). Serum 25(OH)D was measured using an enzyme-linked immunosorbent assay (Immunodiagnostic Systems Limited, Boldon, UK). All interassay and intra-assay coefficients of variation were less than 10% [34].

### 2.4. Statistical Analysis

Data that were normally distributed were expressed as mean ± SD, while data that did not follow a normal distribution were expressed as median (range). Normal and nonnormal distributions between groups were compared using Student's *t*-test and Wilcoxon rank sum test, respectively. We used correlation coefficients, linear regression, and logistic regression to assess relationships. Statistical significant was set at *P* < 0.05 (two-sided).

## 3. Results

Table 1 shows the demographic data and biochemical parameters. Among the 307 hospitalized patients, 102 were males and 205 were females. The mean age of the study population was 52.2 ± 14.6 years. Among the patients, 237 (77.2%) had symptomatic PHPT, 138 (45.0%) had anemia, and 77 (25.1%) had hypomagnesemia.

We compared the parameters between the nonanemic and anemic group by sex (Table 2). A total of 138 patients (49 males (48.0%) and 89 females (43.4%)) had anemia, accounting for 45.0% of the total population. Average serum magnesium was significantly lower in the anemic group than in the nonanemic group in both males and females (0.75 ± 0.17 vs. 0.88 ± 0.19 mmol/L, *P* < 0.05; 0.80 ± 0.19 vs. 0.89 ± 0.16 mmol/L, *P* < 0.05, respectively). Furthermore, average albumin and eGFR levels were significantly lower in the anemic group than in the nonanemic group in both males and females. In contrast, average creatinine, PTH, and corrected calcium levels were significantly higher in the anemic group. Older patients and patients with comparatively lower BMI values were associated with anemia in females. Similarly, there was a trend in males, but the result was not statistically significant. Our data showed no statistically significant differences in serum phosphorus and 25(OH)D levels between the groups with and without anemia.

**Figure 1:**
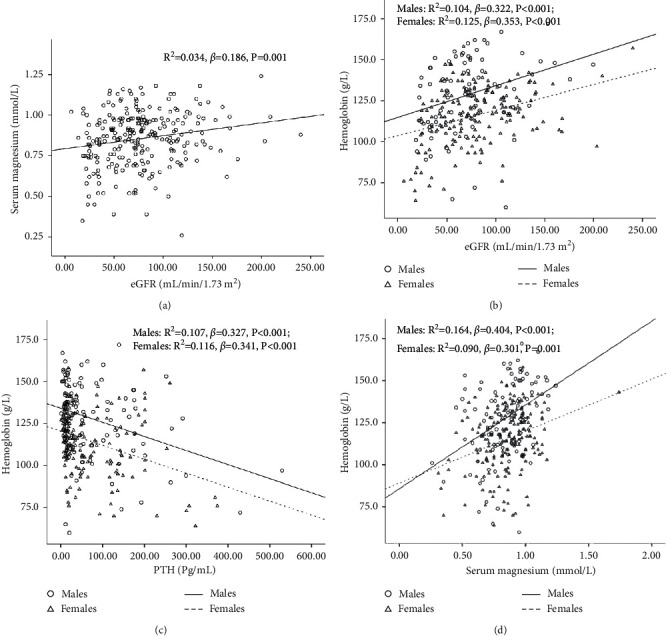
Relationship between serum hemoglobin/magnesium and various indicators in patients with PHPT.

General regression analysis showed that serum magnesium was positively correlated with eGFR (*P* < 0.05; Figure 1(a)). eGFR and serum magnesium were positively correlated with hemoglobin (*P* < 0.05; Figures 1(b) and 1(d)). On the contrary, serum PTH was negatively correlated with hemoglobin (*P* < 0.05; Figure 1(c)). Consistently, this correlation remained significant after the adjustment of demographic data including age, sex, disease duration, and BMI (Table 3, Model 2). Serum albumin and calcium were correlated in both males and females (Table 2). After adjusting these indexes, the association between hemoglobin and PTH/magnesium remained significant (Table 3, Model 3). eGFR and PTH were correlated with hemoglobin; therefore, they were further selected as confounding factors for the multivariate regression analysis. High serum PTH and low serum magnesium were associated with low hemoglobin, independent of eGFR, PTH, or magnesium levels (Table 3, Models 4-5).

Our findings revealed that hypomagnesemia was associated with lower hemoglobin levels, independent of age, sex, disease duration, BMI, albumin, calcium, eGFR, and PTH in PHPT patients.

## 4. Discussion

The incidence of hypomagnesemia is approximately 2% in the general population [35]. In this study, hypomagnesemia had a high prevalence rate in PHPT patients (77/307; 25.1%). Magnesium is maintained within a normal range by a dynamic interplay among the intestines (absorption), bone (deposition), and kidneys (excretion), and disturbances in these organs may contribute to hypomagnesemia [36, 37]. Hypomagnesemia has been observed in PHPT patients, especially in association with high serum calcium levels or kidney damage [28–30]. High prevalence of hypomagnesemia in patients with CKD reveals that impairment in renal function may affect the absorption of magnesium and lead to hypomagnesemia [25, 38]. As seen in our study, serum magnesium was positively correlated with eGFR. Furthermore, hypercalcemia can result in hypomagnesemia by increased filtered calcium load to the loop of Henle, resulting in decreased reabsorption of magnesium [39]. The high prevalence of hypomagnesemia might be the result of our patient population having higher serum calcium levels, severe bone disease, and kidney stones. Our previous study showed that compared to PHPT patients from the USA, PHPT patients from Changsha have higher serum calcium, PTH, and alkaline phosphatase (ALP) levels; lower 25(OH)D levels and bone mineral density; and increased renal stone incidence, suggesting that they have more severe PHPT. It is unclear why there are differences in these parameters between PHPT patients from the USA and China. One possibility is that PHPT patients in the USA are diagnosed at the asymptomatic stage, because most individuals in the USA receive annual physical examination [40, 41]. In China, most PHPT patients are diagnosed when they were hospitalized due to the presence of kidney stones, skeletal lesions, or other symptoms related to hypercalcemia [42]. Other possible explanations include differences in ethnicity and nutritional status [43].

In this study, we assessed the prevalence of anemia and associations between biochemical indices of disease severity and anemia in PHPT patients. A total of 138 patients (49 males (48.0%) and 89 females (43.4%)) had anemia, accounting for 45.0% of the total population. Older patients and patients with lower BMI values were associated with anemia. This phenomenon was also observed in the elderly patient population [44]. The association between serum albumin and anemia may be due to a reduction in hepatic protein synthesis as a result of decreased food intake, malnutrition, advanced age, or sarcopenia [45].

There was a significant reduction in hemoglobin levels with decreasing eGFR values as previously reported [46]. The primary cause of anemia is a reduction in EPO synthesis due to loss of renal functional mass [47]. Impaired renal function leads to the accumulation of toxins. Imbalances in calcium/phosphate, acid/base, and electrolytes resulting from impaired renal function affect RBC shape and survival [48]. All these factors are responsible for low hemoglobin levels with poor kidney function.

Conversely, we obtained a significant negative association between PTH and hemoglobin, independent of age, sex, disease duration, BMI, albumin, eGFR, calcium, and magnesium. This finding is consistent with the fact that high PTH in secondary hyperparathyroidism results in anemia probably as a result of erythropoiesis inhibition, marrow fibrosis, and blood loss by reducing platelet aggregation [49].

In addition, we found a significant association between serum magnesium and hemoglobin levels, independent of age, sex, disease duration, BMI, albumin, calcium, eGFR, and PTH. A strong association between serum magnesium and hemoglobin levels has been reported in non-PHPT patients. The results of this study confirm such association in PHPT patients. The mechanism of the relationship between hemoglobin and magnesium is not clear. It is possible that magnesium deficiency causes hemolysis. After four to five weeks on a low-magnesium diet, magnesium levels in rats rapidly decreased and hemoglobin levels significantly decreased compared with the control group. Anemia was accompanied by severe reticulocytosis, reduction in hemoglobin and hematocrit levels, and reduced lifespan and half-life of RBCs. Hemolytic anemia in a state of magnesium deficiency may be caused by energy metabolism disorders in RBCs [21]. In a beta-thalassemia model, magnesium deficiency caused hemolytic anemia, and anemia improved upon magnesium supplementation [50]. In patients with sickle cell anemia, oral magnesium supplementation reduced dense erythrocyte, absolute reticulocyte, and immature reticulocyte counts and improved erythrocyte membrane transport abnormalities [51]. Furthermore, magnesium is the cofactor of several enzymes involved in protein and nucleic acid synthesis. Erythrocyte energy metabolism and hemoglobin synthesis may decrease in magnesium deficiency, thereby resulting in anemia [22]. Researchers have shown that high serum magnesium levels are correlated with an adequate EPO responsiveness in patients undergoing maintenance hemodialysis [24]. In addition, low magnesium levels cause inflammation and endothelial dysfunction [52], which are known risk factors for anemia.

There were some limitations in our study. First, ionized calcium was not assessed. Second, we were unable to obtain data on hemolysis and inflammation markers. Third, we did not include other clinical important risk factors for anemia. Fourth, these findings are related to populations with a severe PHPT. Thus, these relationships cannot be immediately translated to asymptomatic PHPT outpatients. Fifth, the lack of a control group is a weakness of the study. Indeed, we do not have information regarding whether or not this PHPT population had different Mg levels and prevalence of anemia as compared with non-PHPT-matched inpatients. Finally, the conclusions of this study are weakened by its retrospective design.

Despite these limitations, this study had several strengths. First, this study evaluated data from a large cohort of PHPT patients. Second, blood measurements were performed at the same hospital laboratory. Third, we assessed the prevalence of hypomagnesemia in patients with PHPT. Fourth, this is the first study that assessed whether there is any association between serum magnesium and hemoglobin levels in PHPT patients. Finally, we found that hypomagnesemia, which is a frequent electrolyte disorder in PHPT patients, is associated with hemoglobin levels.

In conclusion, hypomagnesemia is a common electrolyte disorder in PHPT patients. Hypomagnesemia is associated with lower hemoglobin, independent of albumin, calcium, eGFR, and PTH in PHPT patients.

## Figures and Tables

**Table 1:** Baseline characteristics of the subjects.

Number of subjects (males/females)	307 (102/205)
Age (years)	52.2 ± 14.6
Duration (years)	4.48 ± 7.06
BMI (kg/m^2^)	22.4 ± 3.4
Hemoglobin (g/L)	120 ± 21
eGFR (mL/min/1.73 m^2^)	76.0 ± 39.1
Creatinine (umol/L)	91.4 ± 76.2
Albumin (g/L)	38.5 ± 7.7
Serum calcium (mmol/L)	2.85 ± 0.44
Corrected calcium (mmol/L)	3.11 ± 0.57
Serum phosphorus (mmol/L)	0.78 ± 0.28
Serum magnesium (mmol/L)	0.85 ± 0.18
PTH (pg/mL)	68.0 ± 80.9
25(OH)D (ng/mL)	33.6 ± 17.3
Symptomatic patients, *n* (%)	237 (77.2)
Anemia, *n* (%)	138 (45.0)
Hypomagnesemia, *n* (%)	77 (25.1)

BMI, body mass index; eGFR, estimate glomerular filtration rate; PTH, parathyroid hormone; 25(OH)D: 25-hydroxyvitamin D.

**Table 2:** Comparison of the parameters between the nonanemia group and the anemia group by sex.

	Males			Females		
	Nonanemia group (*n* = 53)	Anemia group (*n* = 49)	*P*	Nonanemia group (*n* = 116)	Anemia group (*n* = 89)	*P*
Age (years)	46.9 ± 15.5	49.8 ± 16.1	0.368	50.1 ± 14.1	57.3 ± 12.5	<0.001
Duration (years)	4.74 ± 8.68	4.69 ± 6.88	0.472	4.02 ± 5.68	5.21 ± 8.03	0.321
BMI (kg/m^2^)	22.8 ± 2.9	21.7 ± 3.1	0.111	23.5 ± 3.6	21.8 ± 3.4	0.002
Hemoglobin (g/L)	145 ± 11	107 ± 17	<0.001	132 ± 8	104 ± 13	<0.001
eGFR (mL/min/1.73 m^2^)	81.1 ± 39.7	58.4 ± 31.4	0.006	90.4 ± 37.3	69.9 ± 38.9	<0.001
Creatinine (umol/L)	88.8 ± 35.7	140.9 ± 108.3	0.003	62.7 ± 25.5	92.5 ± 89.0	0.001
Albumin (g/L)	39.6 ± 2.8	36.9 ± 4.5	0.002	40.1 ± 4.5	37.5 ± 11.0	0.039
Serum calcium (mmol/L)	2.82 ± 0.33	3.03 ± 0.48	0.011	2.81 ± 0.43	2.79 ± 0.45	0.783
Corrected calcium (mmol/L)	3.03 ± 0.55	3.35 ± 0.50	0.003	2.94 ± 0.49	3.17 ± 0.58	0.004
Serum phosphorus (mmol/L)	0.70 ± 0.19	0.76 ± 0.17	0.240	0.79 ± 0.25	0.81 ± 0.32	0.561
Serum magnesium (mmol/L)	0.89 ± 0.19	0.76 ± 0.17	0.001	0.90 ± 0.17	0.82 ± 0.17	0.001
PTH (pg/mL)	54.2 ± 60.0	114.7 ± 112.8	0.002	45.2 ± 60.4	72.8 ± 80.6	0.006
25(OH)D (ng/mL)	36.7 ± 20.4	32.5 ± 13.3	0.389	33.9 ± 19.2	32.8 ± 15.9	0.705
Hypomagnesemia *n* (%)	10(18.9)	23(47.0)	0.002	10(8.6)	34(38)	0.002

BMI, body mass index; eGFR, estimate glomerular filtration rate; PTH, parathyroid hormone; 25(OH)D, 25-hydroxyvitamin D. Hypomagnesemia was defined as serum magnesium <0.75 mmol/L.

**Table 3:** Association between magnesium/PTH and hemoglobin.

	Magnesium and hemoglobin (*n* = 307)			PTH and hemoglobin (*n* = 307)		
	*β*	*R* ^2^	*P*	*β*	*R* ^2^	*P*
Model 1	0.331	0.094	<0.001	−0.288	0.080	<0.001
Model 2	0.275	0.213	<0.001	−0.257	0.208	<0.001
Model 3	0.265	0.241	<0.001	−0.262	0.243	<0.001
Model 4	0.230	0.272	<0.001	−0.225	0.283	<0.001
Model 5	0.174	0.301	0.004	−0.176	0.301	0.003

Model 1: general regression analysis; Model 2: adjusted for age, sex, duration of disease, and BMI; Model 3: adjusted for age, sex, duration of disease, BMI, albumin, and calcium; Model 4: adjusted for age, sex, duration of disease, BMI, albumin, calcium, and eGFR; Model 5: adjusted for age, sex, duration of disease, BMI, albumin, calcium, eGFR, and PTH.

## Data Availability

The data used and/or analyzed during the present study are available from the corresponding author on reasonable request.
